# The Efficacy of the WeChat App Combined with Pelvic Floor Muscle Exercise for the Urinary Incontinence after Radical Prostatectomy

**DOI:** 10.1155/2020/6947839

**Published:** 2020-03-18

**Authors:** Shen Junwen, Wang Rongjiang

**Affiliations:** Departments of Urology, The First People's Hospital of Huzhou, Zhejiang Province, Zhejiang Huzhou 313000, China

## Abstract

**Objective:**

This research is aimed at studying the effect of the WeChat app combined with pelvic floor muscle exercise (PFME) on urinary incontinence (UI) for patients treated with radical prostatectomy (RP). *Patients and Methods.* We retrospectively reviewed 112 patients who not only had done open RP or laparoscopic RP in our institution but also had sufficient data: preoperative information and more than one year of follow-up records. All the patients received instructions in correct pelvic floor muscle contraction and were encouraged to train the pelvic floor muscle. 58 patients, who were offered additional training guide by the WeChat app after hospital, were divided into group A, while the other 54 patients, who did PFME alone after hospital, were divided into group B. All the patients underwent a 24 h pad test at 3 days, 1 month, 3 months, 6 months, and 12 months after catheter removal. The differences in preoperative information and the results of the 24 h pad test in the follow-up, from the two groups of patients, were compared statistically. And we defined “urinary continence” as 0 g at a 24 h pad test.

**Results:**

No statistically significant difference in background variable in patients of group A and group B was found. On a 24 h pad test (g), group A had better results compared to group B: 254 ± 76 vs 293 ± 86 (1 month, *p* < 0.05), 76 ± 47 vs 98 ± 58 (3 months, *p* < 0.05), 76 ± 47 vs 98 ± 58 (3 months, *p* < 0.05), 76 ± 47 vs 98 ± 58 (3 months, *p* < 0.05), 76 ± 47 vs 98 ± 58 (3 months, *p* < 0.05), 76 ± 47 vs 98 ± 58 (3 months, *p* < 0.05), 76 ± 47 vs 98 ± 58 (3 months,

**Conclusion:**

Compared to doing PFME alone, the WeChat app combined with pelvic floor muscle training can decrease urine leakage and increase the number of urinary continence after radical prostatectomy.

## 1. Introduction

The urinary incontinence, which can be troublesome and has been shown to affect the patients' quality of life, is the most common side effect after the radical prostatectomy (RP). Much effort was made to improve this problem, like surgical method improvements and postoperative rehabilitation. The pelvic floor muscle exercise (PFME) was ameliorated in many types of research to improve the patients' urinary condition after RP. And Overgard et al. [1] made a randomized controlled trial, and this trial proved that physiotherapist-guided PFME could improve the urinary incontinence after RP. They found that more patients would get benefits from the physiotherapist-guided pelvic floor muscle exercise in 3 and 12 months after RP, compared with the patients who did PFME alone. This trial's results were published in the *European Journal*. In our hospital, most patients after RP do PFME alone at home. And we guess WeChat would take the place of physiotherapists to do the patients a favor and improve their urinary incontinence.

WeChat, like Facebook in the USA, is widely used in China. And our institution uses WeChat as a communication tool between doctors, nurses, and patients who had left the hospital. We used WeChat, instead of seeing the patients when they came back to our clinic, to guide the patients to do pelvic floor muscle exercise at home in the past three years.

In order to verify the effect of the WeChat app combined with PFME on urinary incontinence, we made a retrospective study and compared the results of patients who did PFME alone and patients who did PFME following WeChat.

## 2. Methods

### 2.1. Patients

The research was conducted for patients who not only were operated with open radical prostatectomy (RP) or laparoscopic RP in our institution between January 2016 and December 2018 but also had sufficient data of preoperative information and had more than one year of follow-up records. This study was approved by the ethics committee of our hospital, and the standard of inclusion and exclusion criteria was as follows:

*Inclusion criteria:* (1) patients with localized prostate cancer, (2) patients below 80 years old, and (3) patients with urinary continence before the operation.

*Exclusion criteria:* (1) patients diagnosed with neurological diseases or mental diseases, (2) patients that are unable to do PFME, (3) patients diagnosed with chronic obstructive pulmonary disease (COPD), (4) patients with difficultly controlled hypertension, (5) patients with inguinal hernia history, (6) patients that begin to take oral drugs to treat UI during the follow-up phase, and (7) patients that need further treatment for prostate cancer (chemotherapy, radiotherapy, or reoperation).

There were 112 patients who met the standard of the inclusion criteria. All the patients received instructions in correct PFME and were encouraged to train pelvic floor muscle after catheter removal. 58 patients, who were offered additional training guide by the WeChat app after hospital (range of times of training guide by WeChat: 6-51 weeks, mean times: 15 ± 5.7 weeks), were divided into group A, while the other 54 patients, who do PFME alone after hospital, were split into group B. No statistically significant difference in background variable of patients in group A and group B was found (Table 1).

### 2.2. Surgical Technique

The operations were performed on open RP or laparoscopic RP by two urologists who had done open RP and laparoscopic RP in more than 200 cases. The nerve-sparing RP was usually performed in patients with low-grade (Gleason score ≤ 6) and low-stage (T1c) disease. And the nerve-sparing RP would not be considered for the patients with Gleason score ≥ 4 + 3, cancer in bilateral biopsies or preoperative serum prostate-specific antigen (s-PSA) level > 10 ng/ml. The bladder neck was preserved unless there was a palpable tumor at the base of the prostate. In nearly all patients, the apex of the seminal vesicles and the puboprostatic ligaments were preserved.

### 2.3. Intervention

All the participants (groups A and B) were individually informed of the anatomy and function of the pelvic floor muscles and how to correctly contract their muscles. All the patients were instructed to perform three sets of 10 contractions daily after catheter removal, including holding the contraction for 6–8 s and adding three to four fast contractions at the end of each contraction.

WeChat has two main functions for our patients: (1) it contains an instructional video which can instruct patients to perform PFME correctly and (2) it can push the video to patients on a fixed time and remind patients to do PFME automatically. The patients in group A were instructed to perform PFME through watching the instructional video at home, while the patients in group B did PFME alone. Training frequency was recorded on WeChat. The nurses communicated with all the patients (groups A and B) with the condition of urine and PFME by phone or WeChat every week, and nurses made records of the results.

### 2.4. Outcomes

All the patients were examined by the nurse and urologist at 3 days and 1, 3, 6, and 12 months after catheter removal. The primary outcome measure was self-reported continence after surgery. Urinary continence was defined as 0 g for a 24 h pad test (the pad was weighed before and after use). We assessed the degree of urinary incontinence based on the 24 h pad test.

### 2.5. Statistical Analyses

SPSS19.0 statistical software was used to process the data, and a *T*-test was adopted for calculation and data comparison and a *χ*^2^ test for the enumeration of data comparison, where *p* < 0.05 was defined as the statistically significant difference.

## 3. Results

On the 24 h pad test, the results of the patients in group A were 476 ± 132 g (3 days), 254 ± 76 g (1 month), 76 ± 47 g (3 months), 23 ± 31 g (6 months), and 5 ± 3 g (12 months). The improvement (the consequence of 12 months − the consequence of 3 days) was 460 ± 119 g. The numbers of patients who recovered from being urinary continent were 4 (1 month, 7%), 18 (3 months, 31%), 29 (6 months, 50%), and 40 (12 months, 69%), while the results of the patients in group B on the 24 h pad test were 513 ± 148 g (3 days), 513 ± 148 g (1 month), 98 ± 58 g (3 months), 48 ± 41 g (6 months), and 11 ± 5 g (12 months). The improvement was 485 ± 125 g. The numbers of patients in group B who recovered from being urinary continent were 1 (1 month, 2%), 7 (3 months, 13%), 13 (6 months, 24%), and 34 (12 months, 63%). On 24 h pad test (g), group A had better results compared to group B: 254 ± 76 vs 293 ± 86 (1 month, *p* < 0.05), 76 ± 47 vs 98 ± 58 (3 months, *p* < 0.05), 23 ± 31 vs 48 ± 41 (6 months, *p* < 0.001), and 5 ± 3 vs 11 ± 5 (12 months, *p* < 0.001), respectively. On the urinary continence, 31% were continent in group A vs 13% in group B (3 months, *p* < 0.05), and it increased to 50% in group A compared to 24% in group B (6 months, *p* < 0.01). Other results found no statistically significant difference (Table 2).

## 4. Discussion

Postoperative incontinence is still a common problem following surgery for prostate cancer, even we used newer techniques such as robotic-assisted laparoscopic prostatectomy (RALP). It was reported that the incidence rate of UI reaches 8-87% on the sixth month and 5-44.5% on the twelfth month postoperatively [2–5]. Recently, a prospective, controlled [3], non-RCT of patients undergoing RP in 14 centers using RALP or radical retropubic prostatectomy (RRP) was published. 21.3% of the patients after RALP and 20.2% of the patients after RRP were incontinent at twelve months after the operation. Because of a different definition of urinary continence, the range of the rates of urinary continence after RP in the above researches was quite big. And we defined urinary continence as 0 g for the 24 h pad test, which was a strict standard for urinary continence; there were 69% patients in group A and 63% patients in group B who recovered urinary continence at twelve months after the operation in our research.

What facts are associated with urinary incontinence after RP? There have been many researchers trying to explain the reasons. Zachovajeviene et al. [6] evaluated the dynamics of pelvic floor muscle strength and endurance and urinary incontinence in a 6-month period in men after radical prostatectomy, and they found that pelvic floor muscle strength caused a more significant decrease in urinary incontinence than endurance. Pastore et al. [7] did a systematic review and proved that persistent detrusor overactivity had an essential role in the urinary incontinence. Many systematic reviews [8–10] verified that RALP might decrease the time of recovering for urine after RP; there was still about 5-27.8% of patients who had various degrees of urinary incontinence 12 months after RALP.

Whatever facts are causing urinary incontinence after RP, PFME is still recommended as a prior conservation treatment. Mungovan et al. [11] made a prospective analysis and discovered that patients with various degrees of urinary incontinence had benefited from PFME and regular and standard PFME would help patients to have an earlier recovery of urine. In order to improve the recovery of urine after RP, quite a few researchers, like Santa Mina et al. [12], tried to change the frequency and degree of PFME. Other researchers suggested our patients should do PFME under physiotherapist-guided or ultrasound-guided [13] methods.

The topic of whether physiotherapist-guided PFME can improve the urinary incontinence after RP was widely discussed several years ago. The supports from studies like Overgard et al. [1] affirmed the value of this method, while the opponents like Nissen et al. [14] claimed no statistical difference in the urinary continence after RP was found between the patients who got help from physiotherapist and those who did not. However, the shorter time of urinary incontinence after RP appeared in the patients who got help from a physiotherapist in Nissen's research. And Dubbelman et al. [15] considered physiotherapist-guided pelvic floor muscle exercise as a low-cost efficiency method, because this therapeutic method would take too many medical resources and most patients could not complete this therapeutic method more than 6 months.

WeChat may have the chance to solve the above problem and give our patients a better improvement for their urinary continence after RP. Firstly, WeChat had been widely used in China, and the app would remind our patients to do the PFME three times a day. Obviously, WeChat would save the time of patients who no longer needed to come back to our hospital for physiotherapist-guided training. And the reminder may increase the patients' frequency of PFME. Secondly, the patients who followed the instruction and rhythm on WeChat were more comfortable to do the pelvic floor muscle exercise correctly than doing it alone. Thirdly, WeChat is free and it saves the cost of patients, improving cost efficiency.

In our research, the patients of group A had better results on the number of urinary continence and had less urine leakage in the 24 h pad test. And the patients had done PFME more times after we compared the results from the nurses' weekly follow-up records. Our experience was that it would benefit not only our patients in the recovery but also the doctors and nurses to save our time and increase our efficiency, provided that we can use this new mobile app properly.

## 5. Conclusions

Does the WeChat app combined with pelvic floor muscle exercise reduce the urinary incontinence after radical prostatectomy? Our research had proved the merit of this new method. It increased the number of patients who recovered from urinary continence after PR and decreased urine leakage. Considering it is free and convenient, the WeChat app combined with pelvic floor muscle exercise deserves to be used in more patients.

## Figures and Tables

**Table 1:** Background variable of patients in group A and group B.

Variable	Group A *N* = 58	Group B *N* = 54	*p* value
Age (years), mean ± SD	61 ± 4.1	59 ± 5.7	*p* > 0.05
Body mass index, mean ± SD	26.1 ± 3.0	26.4 ± 3.4	*p* > 0.05
T-PSA lever (ng/ml), mean ± SD	13.8 ± 2.7	14.4 ± 3.2	*p* > 0.05
The operation method, *n*			
Open RP	11	9	
Laparoscopic RP	47	45	*p* > 0.05
Biopsy Gleason score, mean ± SD	6.8 ± 1.2	6.5 ± 1.3	*p* > 0.05
Clinical tumor stage, *n*			
T1c	22	24	*p* > 0.05
T2	31	26	
T3	5	4	
Pathological stage, *n*			
pT2a	8	5	*p* > 0.05
pT2b	15	16	
pT2c	30	29	
pT3a	4	3	
pT3b	1	1	
Surgical margin apex, *n*			
Negative apex	49	48	*p* > 0.05
Positive apex	9	6	
Prostate volume (ml), mean ± SD	37.2 ± 4.9	45.6 ± 5.8	*p* > 0.05

**Table 2:** The results of a 24 h pad test and urinary condition at 3 days, 1 month, 3 months, 6 months, and 12 months after catheter removal (urinary continence was defined as 0 g at a 24 h pad test).

		3 days	1 month	3 months	6 months	12 months	Improvement (the result of 12 months − the result of 3 days)
Group A	24 h pad test (g), mean ± SD	476 ± 132	254 ± 76	76 ± 47	23 ± 31	5 ± 3	460 ± 119
Group B		513 ± 148	293 ± 86	98 ± 58	48 ± 41	11 ± 5	485 ± 125
	*p* value	*p* > 0.05	*p* < 0.05	*p* < 0.05	*p* < 0.001	*p* < 0.001	*p* > 0.05
Group A	Continent, *n*(%)	0	4(7%)	18(31%)	29(50%)	40(69%)	
Group B		0	1(2%)	7(13%)	13(24%)	34(63%)	
	*p* value	*p* > 0.05	*p* > 0.05	*p* < 0.05	*p* < 0.01	*p* > 0.05	

## Data Availability

The data used to support the findings of this study are found in the article.
